# Association of Metabolites with Obesity and Type 2 Diabetes Based on *FTO* Genotype

**DOI:** 10.1371/journal.pone.0156612

**Published:** 2016-06-01

**Authors:** Yeon-Jung Kim, Heun-Sik Lee, Yun Kyoung Kim, Suyeon Park, Jeong-Min Kim, Jun Ho Yun, Ho-Yeong Yu, Bong-Jo Kim

**Affiliations:** Division of Structural and Functional Genomics, Center for Genome Science, Korea National Institute of Health, Chungcheongbuk-do, Korea; Medical Clinic, University Hospital Tuebingen, GERMANY

## Abstract

The single nucleotide polymorphism rs9939609 of the gene *FTO*, which encodes fat mass and obesity–associated protein, is strongly associated with obesity and type 2 diabetes (T2D) in multiple populations; however, the underlying mechanism of this association is unclear. The present study aimed to investigate *FTO* genotype–dependent metabolic changes in obesity and T2D. To elucidate metabolic dysregulation associated with disease risk genotype, genomic and metabolomic datasets were recruited from 2,577 participants of the Korean Association REsource (KARE) cohort, including 40 homozygous carriers of the *FTO* risk allele (AA), 570 heterozygous carriers (AT), and 1,967 participants carrying no risk allele (TT). A total of 134 serum metabolites were quantified using a targeted metabolomics approach. Through comparison of various statistical methods, seven metabolites were identified that are significantly altered in obesity and T2D based on the *FTO* risk allele (adjusted *p* < 0.05). These identified metabolites are relevant to phosphatidylcholine metabolic pathway, and previously reported to be metabolic markers of obesity and T2D. In conclusion, using metabolomics with the information from genome-wide association studies revealed significantly altered metabolites depending on the *FTO* genotype in complex disorders. This study may contribute to a better understanding of the biological mechanisms linking obesity and T2D.

## Introduction

Obesity and type 2 diabetes (T2D) are complex disorders that present a major public health problem worldwide. Thus, there is an urgent need to identify the risk factors for obesity and T2D, as their prevalence continues to increase in many countries. Obesity is defined as a metabolic disorder caused by a hypercaloric diet and/or malnutrition that results in increased accumulation of abnormal body fat and raises the risk of many chronic diseases, including T2D, cardiovascular disease, and cancer [1]. Recently, genome-wide association studies (GWAS) have identified a number of genetic polymorphisms that are associated with an increased risk for obesity and T2D [2–4]. The contribution of most gene polymorphisms to the variability within an organismal phenotype appears to be a small. Nevertheless, several genetic polymorphisms have a substantial impact on the risk of obesity and T2D. Variants in the fat mass and obesity–associated gene *FTO* have been identified as the strongest common genetic risk factors for obesity and T2D. The first reported association of an *FTO* variant with obesity and T2D was for variant rs9939609 in a European population [5]. Since then, the genetic association of variant rs9939609 with obesity and T2D has been demonstrated in a Korean population based on GWAS results [6, 7], and other studies have confirmed that the association of *FTO* variants with obesity or T2D is not dependent on ethnic background [8–10]. However, the molecular and cellular mechanisms underlying this association still need elucidation.

Metabolites are small molecules of diverse biochemical properties that can be measured in body fluids such as blood, serum, and urine. Analyzing metabolites provides a functional readout of the physiological state of phenotypes and enables the discovery of previously undetected biological mechanisms underlying diseases and metabolic pathways [11]. Recently GWAS have been demonstrated to investigate the genetic influences on all metabolites traits (mGWAS) [12]. mGWAS is an effective tool for identifying genes associated with metabolites, and offers an opportunity to infer novel biological mechanisms underlying the association between single nucleotide polymorphisms (SNPs) and metabolites. Xie *et al*. used this method to demonstrate that genetic variants influencing circulating levels of metabolites in the glycine and glutathione pathway are associated with T2D [13]. Several studies have followed a different approach by analyzing selected genes identified from previous studies rather than analyzing the whole genome [14–16]. Then *et al*. reported metabolic alterations in carriers of a common *TCF7L2* variant in a European population [15].

In this study, we identified metabolites significantly associated with obesity and T2D based on *FTO* genotype in 2,577 individuals from the KARE cohort.

## Research Design and Methods

### Ethics statement

All human investigation was conducted according to principles expressed in the Declaration of Helsinki. Written informed consent was given by each participant. The KARE study, including the protocols for subject recruitment, assessment, and obtaining informed consent from participants, was reviewed and approved by an ethics committee (Korea Centers for Disease Control and Prevention Institutional Review Board).

### Study subjects and sampling

The KARE cohort is a community-based cohort assembled through the Korean Genome and Epidemiology Study (KoGES), for which 10,038 initial samples were collected in rural (Ansung) and urban (Ansan) areas near Seoul, South Korea, from 2001 to 2002. Four surveys were subsequently conducted with 29,471 participants who were examined every 2 years from 2003 to 2010. Survey 2 (KARE S2) included 7,515 participants examined from 2005 to 2006. More than 260 traits were examined through epidemiological surveys, physical examinations, and laboratory tests. In total, 2,577 subjects with both genomic and metabolic datasets were recruited from the KARE S2 for this study. Subjects were fast overnight for at least 8 hours before collection of blood sample. In order to minimize dietary diversity effects in metabolomics data, a brief description of food, alcohol, smoking, and nutrient consumption during the fasting conditions should be included in clinical and epidemiological data for subject selection. Individuals with known T2D, morbid obesity, and server disease by physician-validated self-reporting were excluded to avoid potential metabolic influence from pharmacological treatment.

### Genotyping

Genotype data from 10,004 KARE subjects were obtained using the Affymetrix Genome-Wide Human SNP Array 5.0 (Affymetrix, Santa Clara, CA, USA). Quality-control filtering of the genotype data was performed as described by Cho *et al*. [6]. Samples were excluded based on a missing call rate of > 4%, heterozygosity > 30%, gender incompatibility, or cancer. SNPs were excluded based on a missing genotype call rate of > 5%, minor allele frequency < 0.01, and Hardy-Weinberg equilibrium *p* value < 1.00E–6. A total of 8,842 individuals and 352,228 SNPs were included after quality-control analyses. For the present study, the genotype data for 2,577 of 8,842 individuals were used for the genetic association study of metabolic traits. Based on the *FTO* rs9939609 allele, this group included 40 homozygotes with two risk alleles (AA), 570 heterozygous carriers (AT), and 1,967 homozygotes carrying no risk allele (TT).

### Metabolite quantification

Serum metabolite quantification for the 2,577 subjects studied for genotyping was carried out by targeted metabolomics using the AbsoluteIDQ p180 kit (Biocrates Life Sciences, Innsbruck, Austria) containing 40 acylcarnitines, 21 amino acids, 19 biogenic amines, 15 sphingolipids, 90 glycerophospholipids, and 1 hexose (S1 Table). This kit enables simultaneous quantification of the metabolites by liquid chromatography and flow injection analysis mass spectrometry. Pooled health normal human serum as a reference standard was repeatedly quantified 36 times in randomly selected position on the kit to estimate reproducibility. To ensure data quality, each metabolite had to meet the following three criteria: (1) the coefficient of variance (CV) for the metabolites in the reference standards is < 25%; (2) 50% of the measured metabolite concentrations in the reference standards is above the limit of detection, which was set to 3 times the median of the 3 blank samples within each kit; and (3) 50% of the measured metabolite concentrations in the experimental samples is above the limit of detection. In total, 52 metabolites were excluded, leaving 134 for analysis, including 12 acylcarnitines, 21 amino acids, 10 biogenic amines, 12 sphingolipids, 78 glycerophospholipids, and 1 hexose (S1 Table). The concentrations of all analyzed metabolites are reported in μM units.

### Statistics

Statistical analyses were performed to identify metabolites significantly associated with obesity and T2D based on the *FTO* risk allele. All participants were divided into two groups based on *FTO* genotype: risk allele carriers (coding 1) and non-carriers (coding 0). Differences in baseline anthropometric and clinical data between the two groups were assessed using Wilcoxon rank-sum tests. The concentration of each metabolite was log-transformed and normalized using *z* scores by a mean of 0 and a standard deviation of 1. Multivariable-adjusted regression models were calculated to select metabolites associated with obesity and T2D based on genotype. The analysis pipeline for this study was as follows. First, the associations between genotype effect and metabolites were investigated using linear regression analysis adjusted for age and gender. Second, the associations between metabolites and phenotypes—including obesity and T2D—were examined using linear regression analysis adjusted for age and gender. Next, linear regression models were applied with the metabolites as independent variables and body mass index (BMI) values as dependent variables for obesity. The BMI method correlates well with body fat and is the most popular obesity index for clinical practice, health examinations, and surveys in adults [3, 4, 17–19]. For T2D, fasting glucose (Glu0) and 2-h glucose (Glu120) values were used as dependent variables. Finally, common metabolites involved in obesity and T2D based on *FTO* genotype were selected. To handle false discovery rates (type I errors) obtained from multiple comparisons, the cutoff point for significance was adjusted according to the Benjamini-Hochberg procedure and set at a *p* value of 0.05 [20]. Statistical analyses were performed using the statistical R package, version 3.1.2 (http://www.r-projrct.org/).

## Results

### Subject characteristics

Fasting levels of 134 plasma metabolites were obtained from 610 minor allele (A) carriers and 1,967 major allele (T) carriers. The frequency of the rs9939609 allele (A) in the study population was 0.13. Baseline characteristics of the KARE S2 sample are presented in Table 1. Among these characteristics, weight, BMI, and 2-h glucose values were significantly different between *FTO* genotypes.

**Table 1:** Baseline characteristics of the KARE S2 cohort sample^a^,^b^.

	Wild type (n = 1,967)	Carrier type (n = 610)	*p* value
	TT	TA/AA	
**Gender** (M/F)	936/1,031	281/329	—
**Age** (years)	56.97 ± 9.03	57.48 ± 9.11	0.13
**Height** (cm)	159.73 ± 9.22	158.99 ± 8.95	0.94
**Weight** (kg)	62.38 ± 10.34	63.41 ± 10.42	0.02
**BMI** (kg/m^2^)	24.43 ± 3.23	25.06 ± 3.23	3.4E-05
**Fasting glucose** (mg/dL)	95.37 ± 19.28	97.05 ± 21.69	0.11
**2-h glucose** (mg/dL)	138.81 ± 63.29	146.63 ± 68.91	0.008
**HDL cholesterol** (mg/dL)	43.97 ± 10.35	43.33 ± 9.98	0.91

^a^ Values represent mean ± standard deviation. Differences considered statistically significant *p* < 0.05

^b^ BMI, body mass index; HDL, high-density lipoprotein.

### Identification of metabolites associated with *FTO* genotype

We selected metabolites for which concentration differed according to *FTO* genotype. Based on dominant model of linear regression analysis, 19 metabolites had significantly different concentrations between the two groups (Benjamini-Hochberg-adjusted *p* < 0.05; S2 Table). These associations were independent of age and gender. We also carried out the association study by using an additive model, 14 metabolites (Benjamini-Hochberg adjusted *p* < 0.05) were isolated and found to be identical to the output from the dominant model (S3 Table). However, 5 significant metabolites, hexose, valine, PC aa C40:1, PC ae C38:0, and PC ae C40:2, in the dominant model had marginal statistical significance (*p* = 0.052) under the additive model (S3 Table). This result may be affected by small sample size of the risk allele homozygotes (40 AA in 2,577 subjects). Finally, we selected 19 metabolites associated with *FTO* genotype.

### Identification of metabolites associated with intermediate phenotype of obesity and T2D

We first identified genotype-independent metabolites associated with obesity by linear regression analysis using BMI as the criteria for obesity. As a result, 92 metabolites having a significant association with BMI were selected (adjusted *p* < 0.05; S4 Table). We next performed an analysis of genotype-independent metabolites with T2D using Glu0 and Glu120 levels as the criteria for T2D. Using linear regression analysis, we identified 93 metabolites associated with Glu0 with a high statistical significance (adjusted *p* < 0.05; S5 Table) and 104 metabolites associated with Glu120 (adjusted *p* < 0.05; S6 Table). Among these metabolites, 85 were common to Glu0 and Glu120. These mostly metabolites were not only relevant to choline-containing phospholipid metabolic pathway, but also include 68 of 85 metabolites associated with T2D were identified of common metabolites in obesity.

### Identification of metabolites associated with obesity and T2D based on *FTO* genotype

**Fig 1:**
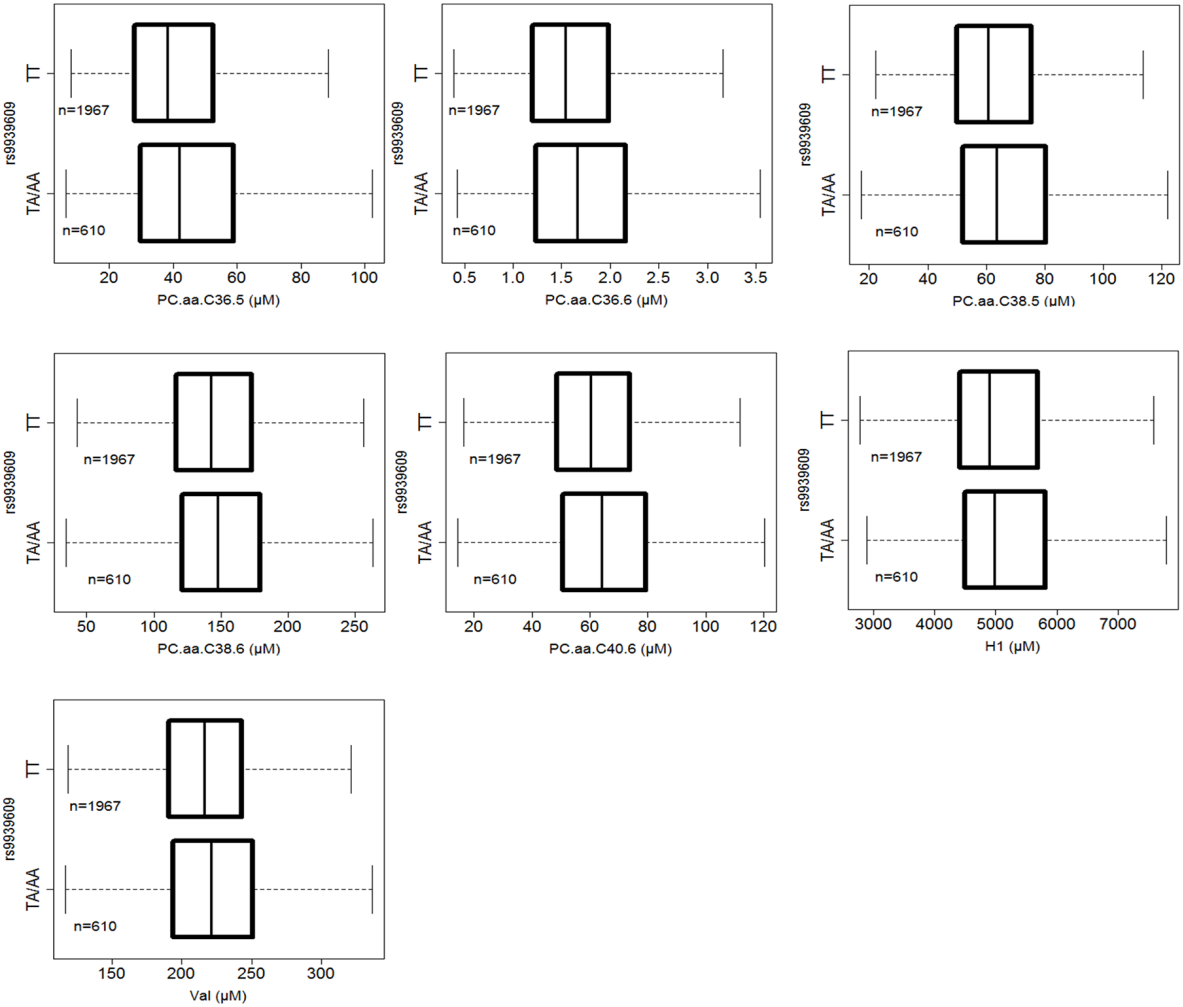
Metabolites displaying significant differences between *FTO* risk allele carriers and non-carriers. They show the differentiation of the population that is induced by these genetically determined metabotypes. Boxes extend from the first to third quartiles, the median is indicated as a horizontal line, and the number of individuals in each group is indicated (n). The *p* values are given in Table 2.

Finally, we identified common metabolites involved in obesity and T2D based on genotype. For seven metabolites, we observed a significant genotype effect on obesity and T2D in *FTO* risk allele carriers compared with control subjects. The seven metabolites included one monosaccharide (hexose), one amino acid (valine), and five glycerophospholipids (specifically, the diacylphosphatidylcholines (PC aa) C36:5, C36:6, C38:5, C38:6, and C40:6). The genotype effects are shown graphically in Fig 1), and the magnitude of each effect and the associated *p* values are shown in Table 2. Among the seven metabolites, the five PCs showed the most pronounced effects. Three of the identified metabolites have been linked with obesity and/or T2D: valine [21–23], hexose [24], PC aa C38:6 [25]. Also, we found that novel metabolites (PC aa C36:5, C38:5, C36:6 and C40:6) contribute to obesity and T2D based on *FTO* genotype.

**Table 2:** Differences in traits of metabolites with a significant *FTO* genotype effect in obesity and T2D.^a^

	Genotype		BMI		Glu0		Glu120	
Metabolite	β-coefficient (95% CI)	adjusted *p*	β-coefficient (95% CI)	adjusted *p*	β-coefficient (95% CI)	adjusted *p*	β-coefficient (95% CI)	adjusted *p*
H1^b^,^c^	0.126 (0.04–0.22)	4.6.E-02	0.72 (0.59−0.84)	1.5E−27	16.68 (16.12–17.24)	0.0E+00	42.08 (39.76−44.39)	1.2E−222
Valine^b^,^c^	0.127 (0.04–0.22)	3.9.E-02	1.04 (0.91−1.16)	5.0E−57	3.74 (2.95–4.53)	5.7E−19	14.71 (12.13−17.30)	4.7E−27
PC aa C36:5	0.175 (0.08–0.27)	1.2.E-02	0.46 (0.34−0.58)	2.3E−12	3.46 (2.70–4.22)	1.1E−17	10.26 (7.75−12.77)	8.3E−15
PC aa C36:6	0.145 (0.06–0.23)	1.7.E-02	0.22 (0.10−0.35)	1.1E−03	1.72 (0.93–2.50)	4.4E−05	5.16 (2.57−7.75)	1.8E−04
PC aa C38:5	0.140 (0.05–0.23)	2.8.E-02	0.34 (0.22−0.47)	1.9E−07	3.00 (2.23–3.76)	1.1E−13	8.28 (5.77−10.79)	4.2E−10
PC aa C38:6^b^	0.154 (0.07–0.24)	1.5.E-02	0.37 (0.24−0.49)	4.4E−08	2.81 (2.03–3.59)	9.5E−12	12.64 (10.10−15.19)	5.6E−21
PC aa C40:6	0.173 (0.08–0.26)	1.2.E-02	0.45 (0.32−0.57)	8.5E−12	1.77 (0.99–2.54)	2.0E−05	9.79 (7.26−12.32)	2.1E−13

^a^ CI, confidence interval; significant association defined by Benjamini-Hochberg-adjusted *p* value < 0.05.

^b^ Known metabolite associated with T2D.

^c^ Known metabolite associated with obesity.

## Discussion

Using GWAS, a number of genetic variants and candidate genes have been reported to be associated with obesity and T2D [2–4]. Although GWAS provide a powerful approach to the discovery of disease-associated gene variants, in most instances there is limited information about the function or molecular mechanisms of the variants identified. Here, we assessed whether GWAS-identified candidate genes were differentially enriched for metabolites associated with obesity and T2D. Using a targeted metabolomics approach, we identified alterations in PC and amino acid metabolism in subjects with potentially increased risk of obesity and T2D as defined by the presence of the rs9939609 risk allele. We identified seven metabolites that were significantly associated with obesity and T2D based on *FTO* genotype.

**Fig 2:**
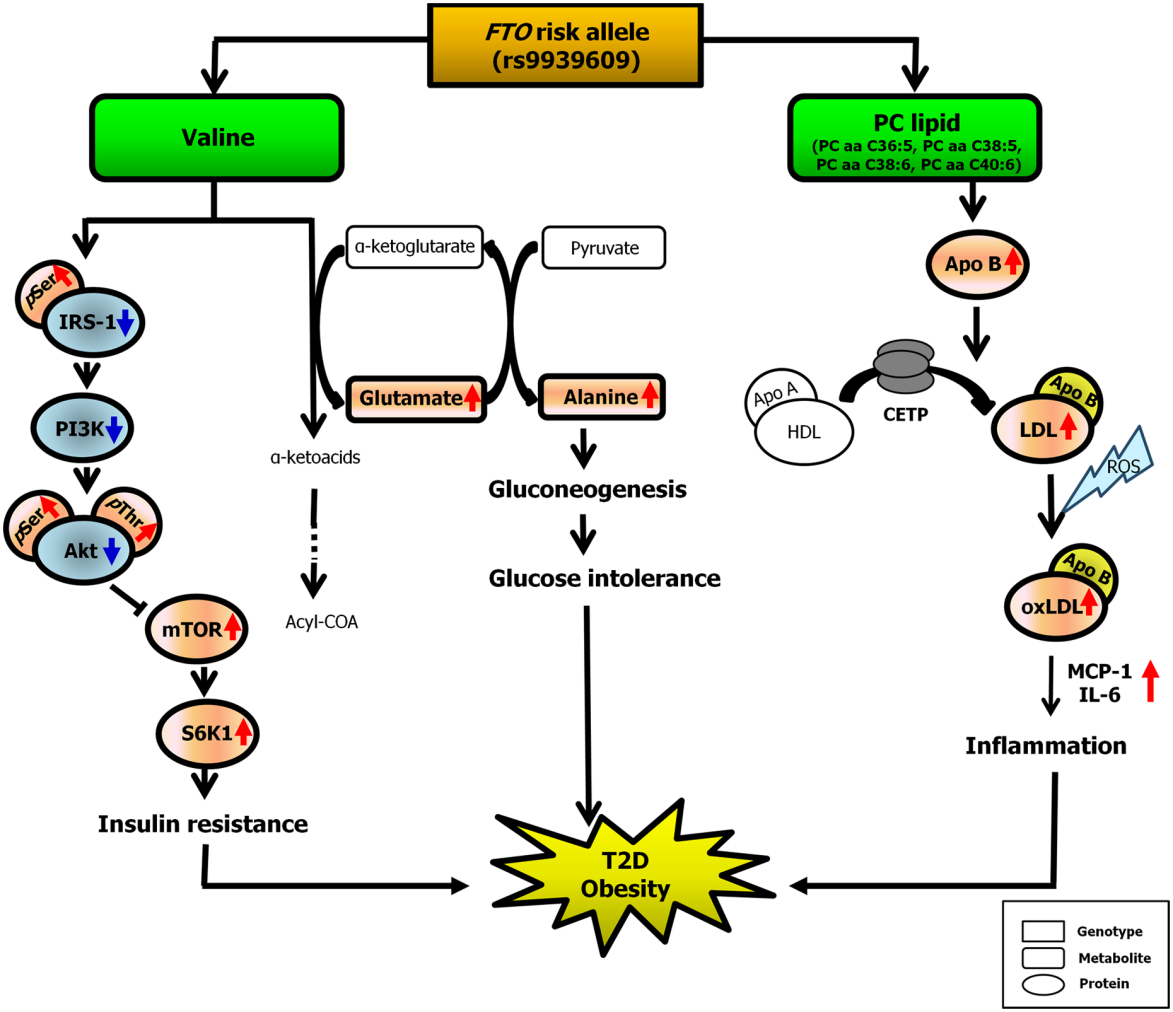
Schematic diagram of metabolic pathways relevant to SNP-metabolite associations. **Left:** PC lipid levels were increased in the rs9939609 risk allele group. Increased PC levels dependent on the *FTO* variant rs9939609 might promote T2D and obesity via fat accumulation in body and by inflammation caused by ApoB-induced LDL augmentation in the blood. **Right:** Valine levels were also increased in the rs9939609 risk allele group. Increased valine levels induce activation of the mTOR/S6K1 kinase pathway and phosphorylation of several serine residues in IRS-1, contributing to insulin resistance. In addition, increased valine catabolic flux may contribute to increased gluconeogenesis and glucose intolerance via glutamate transamination to alanine.

We found a positive association between valine level and the rs9939609 risk allele. Valine is a branched-chain amino acid (BCAA), and alteration of normal BCAA metabolism, leading to elevated blood concentrations of BCAAs and their derivatives, appears to be an early manifestation of insulin resistance [6, 23, 26]. Cahill *et al*. found highly significant correlations between fasting plasma insulin concentration and levels of leucine, isoleucine, and valine in normal and obese individuals [5]. Menge *et al*. reported a significant correlation between plasma valine concentration and the homeostatic model assessment of insulin resistance (HOMA-IR) score [27]. It is not well understood why valine levels are high in individuals with obesity and insulin resistance. One theory is that an increase in BCAA levels activates the mTOR/S6K1 kinase pathway and results in the phosphorylation of several serine residues in IRS-1, contributing to insulin resistance [27, 28]. An increase in BCAAs in muscle cells results in activation of mTOR, impaired insulin-stimulated phosphorylation of Akt/protein kinase B, and reduced insulin-stimulated glucose uptake [3, 28, 29]. Another theory is that a high rate of circulation BCAAs and accumulation of glutamate may increase transamination of pyruvate to alanine. Increases in alanine, a highly gluconeogenic amino acid, could contribute to development of glucose intolerance in obese subjects [23] (Fig 2 Left).

PC is physiologically important as the principal component of eukaryotic cellular membranes [30], as a precursor of signaling molecules [18], and as a key element in lipoproteins [31], bile [18], and lung surfactant [19]. Among of the five PC metabolites associated with obesity and T2D based on *FTO* genotype, four (PC aa C36:5, C38:5, C38:6, and C40:6) are associated with apolipoprotein B (ApoB) [9]. ApoB is the primary apolipoprotein of chylomicrons, low-density lipoprotein (LDL) particles, and very low-density lipoprotein (VLDL) particles, which are responsible for carrying fat molecules, including cholesterol, in circulating blood to all cells. ApoB-containing LDL is transferred to mature high-density lipoprotein (HDL) by the action of cholesteryl ester transfer protein [32]. The generation of reactive oxygen species within blood vessels results in oxidation of lipid components of LDL, generating oxidized LDL. Oxidized LDL activates circulating monocytes, increasing their ability to infiltrate the vascular wall. Consequently, oxidized LDL induces inflammatory responses in blood vessels that lead to atherogenesis [25]. Recent studies suggest that increased lipid oxidation is also associated with T2D and obesity via inflammation [33, 34]. In our study, concentrations of LDL and triglycerides were significantly elevated in the *FTO* carrier group compared with the control group (LDL: 123.6 ± 31.69 vs. 121 ± 31.82 mg/dL, *p* < 0.05; triglycerides: 139.3 ± 72.51 vs. 132.21 ± 67.67 mg/dL, *p* < 0.05; S1 Fig), whereas HDL levels were similar between groups. Thus, it is possible that increased PC levels associated with the rs9939609 variant promote T2D and obesity via fat accumulation in the body as well as by inflammation caused by ApoB-induced LDL augmentation in the blood (Fig 2 Right).

One of the five PC metabolites, PC aa C38:5, is associated with a number of inflammation markers and adipokines associated with increased obesity and insulin resistance, including C-reactive protein (CRP) and resistin [35]. CRP is an acute-phase reactant that rises within hours of the onset of inflammation. It binds to phosphatidylcholine expressed on the surface of dead cells in order to activate the complement system [36]. CRP is strongly linked to cardiovascular disease [37] as well as to components of metabolic syndrome, including insulin resistance, although a recent study suggested that obesity is the major determinant of the CRP/insulin resistance relationship [38, 39].

There are a number of limitations in our study. First, BMI cannot discriminate between fat and lean mass, and they do not reflect body fat distribution. However, BMI, percent body fat, and trunk fat are typically highly correlated [40], and at least one study suggested that they are similarly associated with obesity-related biomarkers and metabolic syndrome. Second, our findings need to be confirmed using another independent population. Third, because we could not recruit sufficient numbers of subjects with the homozygous risk allele genotype, our results need to be verified with a greater number of subjects. Fourth, the biochemical mechanisms leading to the observed changes in metabolite concentrations, as well as their biological significance, require further investigation.

In conclusion, this study provides evidence for changes in phospholipid and amino acid metabolism that may be linked to obesity and T2D in *FTO* risk allele carriers. These data may contribute to a better understanding of the biochemical networks underlying the development of obesity and T2D in individuals carrying the *FTO* risk allele.

## Supporting Information

S1 Fig Concentration of LDL-cholesterol, triglycerides (TG), and HDL-cholesterol between *FTO* risk allele carriers and non-carriers. (**A**) A significant increase in LDL was observed in rs9939609 carriers (TA/AA) compared with non-carriers (TT; *p* < 0.05). (**B**) TG levels displayed the strongest difference between genotype groups (*p* < 0.05). (**C**) There was no significant difference in the concentration of HDL (*p* = **0.91**).
(TIF)

S1 Table Characteristics of the 186 targeted metabolites. (PDF)

S2 Table Identified metabolites association with carriers of *FTO* rs9939609 (TA/AA genotype) by using dominant model. Significant association defined by Benjamini-Hochberge adjusted *p* < 0.05.^a^
(PDF)

S3 Table Identified metabolites association with *FTO* genotype, 40 homozygotes with two risk alleles (AA), 570 heterozygous carriers (AT), and 1,967 homozygotes carrying no risk allele (TT), by using additive model. (PDF)

S4 Table Identified metabolites association with risk of obesity (BMI) in KARE S2 (Significant association defined by Benjamini-Hochberge adjusted *p* < 0.05).^a^ (PDF)

S5 Table Identified metabolites association with risk of T2D (Glu0) in KARE S2 (Significant association defined by Benjamini-Hochberge adjusted *p* < 0.05).^a^ (PDF)

S6 Table Identified metabolites association with risk of T2D (Glu120) in KARE S2 (Significant association defined by Benjamini-Hochberge adjusted *p* < 0.05).^a^ (PDF)
