# Supplementary figures and images for: Association of Metabolites with Obesity and Type 2 Diabetes Based on FTO Genotype
Source: PLoS One. 2016 Jun 1;11(6):e0156612. doi: 10.1371/journal.pone.0156612 (PMC4889059; doi:10.1371/journal.pone.0156612)

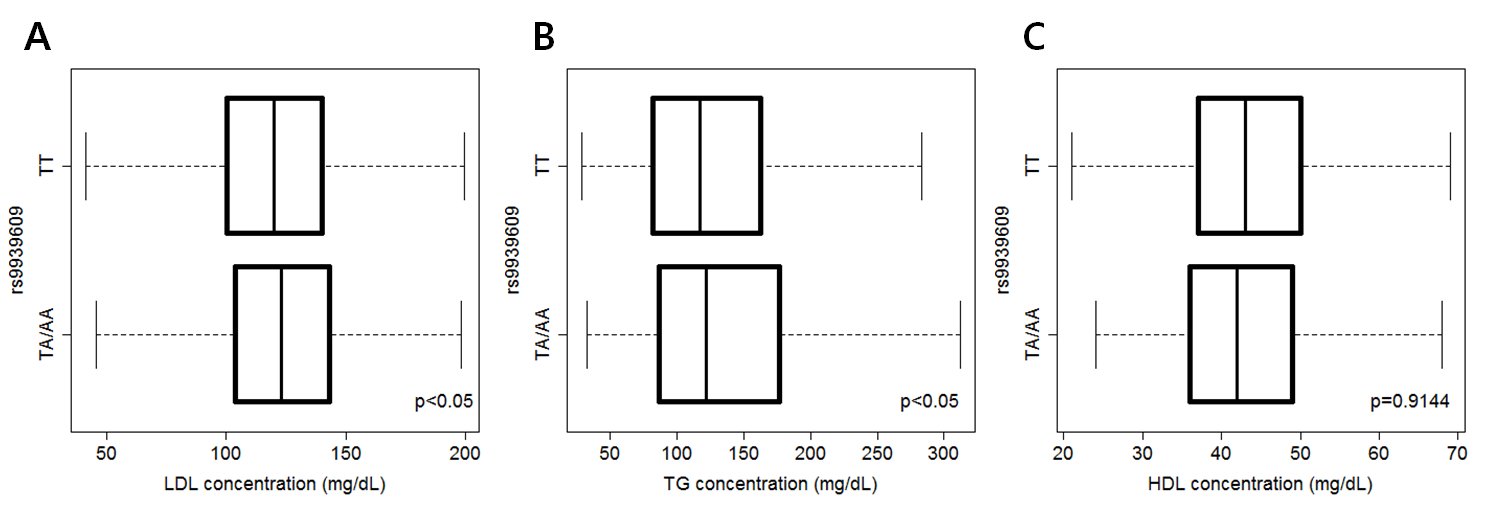

Supplement: S1 Fig — (A) A significant increase in LDL was observed in rs9939609 carriers (TA/AA) compared with non-carriers (TT; p < 0.05). (B) TG levels displayed the strongest difference between genotype groups (p < 0.05). (C) There was no significant difference in the concentration of HDL (p = 0.91). (TIF) [file pone.0156612.s001.tif]
