# Supplementary material for: Association of Metabolites with Obesity and Type 2 Diabetes Based on FTO Genotype
Source: PLoS One. 2016 Jun 1;11(6):e0156612. doi: 10.1371/journal.pone.0156612 (PMC4889059; doi:10.1371/journal.pone.0156612)
Supplement: S1 Table — (PDF) [file pone.0156612.s002.pdf]

S1 Table. Characteristics of the 186 targeted metabolites.

#	MetIDQ Short Name	Biochemical Name	Application
	**Acylcarnitines (12/40)**		
1	C0	Carnitine	Used
2	C2	Acetylcarnitine	Used
3	C3	Propionylcarnitine	Used
4	C3:1	Propenoylcarnitine	Excluded
5	C3-OH	Hydroxypropionylcarnitine	Excluded
6	C4	Butyrylcarnitine	Used
7	C4:1	Butenylcarnitine	Excluded
8	C3-DC (C4-OH)	Hydroxybutyrylcarnitine	Excluded
9	C5	Valerylcarnitine	Used
10	C5:1	Tiglylcarnitine	Excluded
11	C5:1-DC	Glutaconylcarnitine	Excluded
12	C5-DC (C6-OH)	Glutarylcarnitine	Excluded
13	C5-M-DC	Methylglutarylcarnitine	Excluded
14	C5-OH (C3-DC-M)	Hydroxyvalerylcarnitine	Excluded
15	C6 (C4:1-DC)	Hexanoylcarnitine	Excluded
16	C6:1	Hexenoylcarnitine	Excluded
17	C7-DC	Pimelylcarnitine	Used
18	C8	Octanoylcarnitine	Excluded
19	C9	Nonaylcarnitine	Excluded
20	C10	Decanoylcarnitine	Excluded
21	C10:1	Decenoylcarnitine	Excluded
22	C10:2	Decadienylcarnitine	Excluded
23	C12	Dodecanoylcarnitine	Excluded
24	C12:1	Dodecenoylcarnitine	Excluded
25	C12-DC	Dodecanedioylcarnitine	Excluded
26	C14	Tetradecanoylcarnitine	Excluded
27	C14:1	Tetradecenoylcarnitine	Used
28	C14:1-OH	Hydroxytetradecenoylcarnitine	Excluded
29	C14:2	Tetradecadienylcarnitine	Used
30	C14:2-OH	Hydroxytetradecadienylcarnitine	Excluded
31	C16	Hexadecanoylcarnitine	Used
32	C16:1	Hexadecenoylcarnitine	Excluded
33	C16:1-OH	Hydroxyhexadecenoylcarnitine	Excluded
34	C16:2	Hexadecadienylcarnitine	Excluded
35	C16:2-OH	Hydroxyhexadecadienylcarnitine	Excluded
36	C16-OH	Hydroxyhexadecanoylcarnitine	Excluded
37	C18	Octadecanoylcarnitine	Used
38	C18:1	Octadecenoylcarnitine	Used
39	C18:1-OH	Hydroxyoctadecenoylcarnitine	Excluded
40	C18:2	Octadecadienylcarnitine	Used
	**Amino Acids (21/21)**		
41	Ala	Alanine	Used
42	Arg	Arginine	Used
43	Asn	Asparagine	Used
44	Asp	Aspartate	Used
45	Cit	Citrulline	Used
46	Gln	Glutamine	Used
47	Glu	Glutamate	Used
48	Gly	Glycine	Used
49	His	Histidine	Used
50	Ile	Isoleucine	Used
51	Leu	Leucine	Used
52	Lys	Lysine	Used
53	Met	Methionine	Used
54	Orn	Ornithine	Used
55	Phe	Phenylalanine	Used
56	Pro	Proline	Used
57	Ser	Serine	Used

58	Thr	Threonine	Used
59	Trp	Tryptophan	Used
60	Tyr	Tyrosine	Used
61	Val	Valine	Used
**Biogenic Amines (10/19)**			
62	Ac-Orn	Acetylornithine	Used
63	ADMA	Asymmetric dimethylarginine	Used
64	SDMA	Symmetric dimethylarginine	Excluded
65	total DMA	Total dimethylarginine	Excluded
66	alpha-AAA	alpha-Aminoadipic acid	Excluded
67	Carnosine	Carnosine	Excluded
68	Creatinine	Creatinine	Used
69	Histamine	Histamine	Excluded
70	Kynurenine	Kynurenine	Used
71	Met-SO	Methioninesulfoxide	Excluded
72	Nitro-Tyr	Nitrotyrosine	Excluded
73	OH-Pro	Hydroxyproline	Excluded
74	PEA	Phenylethylamine	Excluded
75	Putrescine	Putrescine	Used
76	Sarcosine	Sarcosine	Used
77	Serotonin	Serotonin	Used
78	Spermidine	Spermidine	Used
79	Spermine	Spermine	Used
80	Taurine	Taurine	Used
**Glycerophospholipids (78/90)**			
81	lysoPC a C14:0	lysoPhosphatidylcholine acyl C14:0	Excluded
82	lysoPC a C16:0	lysoPhosphatidylcholine acyl C16:0	Used
83	lysoPC a C16:1	lysoPhosphatidylcholine acyl C16:1	Used
84	lysoPC a C17:0	lysoPhosphatidylcholine acyl C17:0	Used
85	lysoPC a C18:0	lysoPhosphatidylcholine acyl C18:0	Used
86	lysoPC a C18:1	lysoPhosphatidylcholine acyl C18:1	Used
87	lysoPC a C18:2	lysoPhosphatidylcholine acyl C18:2	Used
88	lysoPC a C20:3	lysoPhosphatidylcholine acyl C20:3	Used
89	lysoPC a C20:4	lysoPhosphatidylcholine acyl C20:4	Used
90	lysoPC a C24:0	lysoPhosphatidylcholine acyl C24:0	Excluded
91	lysoPC a C26:0	lysoPhosphatidylcholine acyl C26:0	Excluded
92	lysoPC a C26:1	lysoPhosphatidylcholine acyl C26:1	Excluded
93	lysoPC a C28:0	lysoPhosphatidylcholine acyl C28:0	Excluded
94	lysoPC a C28:1	lysoPhosphatidylcholine acyl C28:1	Excluded
95	PC aa C24:0	Phosphatidylcholine diacyl C24:0	Excluded
96	PC aa C26:0	Phosphatidylcholine diacyl C26:0	Excluded
97	PC aa C28:1	Phosphatidylcholine diacyl C28:1	Used
98	PC aa C30:0	Phosphatidylcholine diacyl C30:0	Used
99	PC aa C30:2	Phosphatidylcholine diacyl C30:2	Excluded
100	PC aa C32:0	Phosphatidylcholine diacyl C32:0	Used
101	PC aa C32:1	Phosphatidylcholine diacyl C32:1	Used
102	PC aa C32:2	Phosphatidylcholine diacyl C32:2	Excluded
103	PC aa C32:3	Phosphatidylcholine diacyl C32:3	Used
104	PC aa C34:1	Phosphatidylcholine diacyl C34:1	Used
105	PC aa C34:2	Phosphatidylcholine diacyl C34:2	Used
106	PC aa C34:3	Phosphatidylcholine diacyl C34:3	Used
107	PC aa C34:4	Phosphatidylcholine diacyl C34:4	Used
108	PC aa C36:0	Phosphatidylcholine diacyl C36:0	Used
109	PC aa C36:1	Phosphatidylcholine diacyl C36:1	Used
110	PC aa C36:2	Phosphatidylcholine diacyl C36:2	Used
111	PC aa C36:3	Phosphatidylcholine diacyl C36:3	Used
112	PC aa C36:4	Phosphatidylcholine diacyl C36:4	Used
113	PC aa C36:5	Phosphatidylcholine diacyl C36:5	Used
114	PC aa C36:6	Phosphatidylcholine diacyl C36:6	Used
115	PC aa C38:0	Phosphatidylcholine diacyl C38:0	Used
116	PC aa C38:1	Phosphatidylcholine diacyl C38:1	Used
117	PC aa C38:3	Phosphatidylcholine diacyl C38:3	Used
118	PC aa C38:4	Phosphatidylcholine diacyl C38:4	Used
119	PC aa C38:5	Phosphatidylcholine diacyl C38:5	Used
120	PC aa C38:6	Phosphatidylcholine diacyl C38:6	Used

121	PC aa C40:1	Phosphatidylcholine diacyl C40:1	Used
122	PC aa C40:2	Phosphatidylcholine diacyl C40:2	Used
123	PC aa C40:3	Phosphatidylcholine diacyl C40:3	Used
124	PC aa C40:4	Phosphatidylcholine diacyl C40:4	Used
125	PC aa C40:5	Phosphatidylcholine diacyl C40:5	Used
126	PC aa C40:6	Phosphatidylcholine diacyl C40:6	Used
127	PC aa C42:0	Phosphatidylcholine diacyl C42:0	Used
128	PC aa C42:1	Phosphatidylcholine diacyl C42:1	Used
129	PC aa C42:2	Phosphatidylcholine diacyl C42:2	Used
130	PC aa C42:4	Phosphatidylcholine diacyl C42:4	Used
131	PC aa C42:5	Phosphatidylcholine diacyl C42:5	Used
132	PC aa C42:6	Phosphatidylcholine diacyl C42:6	Used
133	PC ae C30:0	Phosphatidylcholine acyl-alkyl C30:0	Used
134	PC ae C30:1	Phosphatidylcholine acyl-alkyl C30:1	Excluded
135	PC ae C30:2	Phosphatidylcholine acyl-alkyl C30:2	Excluded
136	PC ae C32:1	Phosphatidylcholine acyl-alkyl C32:1	Used
137	PC ae C32:2	Phosphatidylcholine acyl-alkyl C32:2	Used
138	PC ae C34:0	Phosphatidylcholine acyl-alkyl C34:0	Used
139	PC ae C34:1	Phosphatidylcholine acyl-alkyl C34:1	Used
140	PC ae C34:2	Phosphatidylcholine acyl-alkyl C34:2	Used
141	PC ae C34:3	Phosphatidylcholine acyl-alkyl C34:3	Used
142	PC ae C36:0	Phosphatidylcholine acyl-alkyl C36:0	Used
143	PC ae C36:1	Phosphatidylcholine acyl-alkyl C36:1	Used
144	PC ae C36:2	Phosphatidylcholine acyl-alkyl C36:2	Used
145	PC ae C36:3	Phosphatidylcholine acyl-alkyl C36:3	Used
146	PC ae C36:4	Phosphatidylcholine acyl-alkyl C36:4	Used
147	PC ae C36:5	Phosphatidylcholine acyl-alkyl C36:5	Used
148	PC ae C38:0	Phosphatidylcholine acyl-alkyl C38:0	Used
149	PC ae C38:1	Phosphatidylcholine acyl-alkyl C38:1	Used
150	PC ae C38:2	Phosphatidylcholine acyl-alkyl C38:2	Used
151	PC ae C38:3	Phosphatidylcholine acyl-alkyl C38:3	Used
152	PC ae C38:4	Phosphatidylcholine acyl-alkyl C38:4	Used
153	PC ae C38:5	Phosphatidylcholine acyl-alkyl C38:5	Used
154	PC ae C38:6	Phosphatidylcholine acyl-alkyl C38:6	Used
155	PC ae C40:1	Phosphatidylcholine acyl-alkyl C40:1	Used
156	PC ae C40:2	Phosphatidylcholine acyl-alkyl C40:2	Used
157	PC ae C40:3	Phosphatidylcholine acyl-alkyl C40:3	Used
158	PC ae C40:4	Phosphatidylcholine acyl-alkyl C40:4	Used
159	PC ae C40:5	Phosphatidylcholine acyl-alkyl C40:5	Used
160	PC ae C40:6	Phosphatidylcholine acyl-alkyl C40:6	Used
161	PC ae C42:0	Phosphatidylcholine acyl-alkyl C42:0	Used
162	PC ae C42:1	Phosphatidylcholine acyl-alkyl C42:1	Used
163	PC ae C42:2	Phosphatidylcholine acyl-alkyl C42:2	Used
164	PC ae C42:3	Phosphatidylcholine acyl-alkyl C42:3	Used
165	PC ae C42:4	Phosphatidylcholine acyl-alkyl C42:4	Used
166	PC ae C42:5	Phosphatidylcholine acyl-alkyl C42:5	Used
167	PC ae C44:3	Phosphatidylcholine acyl-alkyl C44:3	Used
168	PC ae C44:4	Phosphatidylcholine acyl-alkyl C44:4	Used
169	PC ae C44:5	Phosphatidylcholine acyl-alkyl C44:5	Used
170	PC ae C44:6	Phosphatidylcholine acyl-alkyl C44:6	Used
	**Sphingolipids (12/15)**		
171	SM (OH) C14:1	Hydroxysphingomyeline C14:1	Used
172	SM C16:0	Sphingomyeline C16:0	Used
173	SM C16:1	Sphingomyeline C16:1	Used
174	SM (OH) C16:1	Hydroxysphingomyeline C16:1	Used
175	SM C18:0	Sphingomyeline C18:0	Used
176	SM C18:1	Sphingomyeline C18:1	Used
177	SM C20:2	Sphingomyeline C20:2	Excluded
178	SM C22:3	Sphingomyeline C22:3	Excluded
179	SM (OH) C22:1	Hydroxysphingomyeline C22:1	Used
180	SM (OH) C22:2	Hydroxysphingomyeline C22:2	Used
181	SM C24:0	Sphingomyeline C24:0	Used
182	SM C24:1	Sphingomyeline C24:1	Used
183	SM (OH) C24:1	Hydroxysphingomyeline C24:1	Used
184	SM C26:0	Sphingomyeline C26:0	Excluded

185	SM C26:1	Sphingomyeline C26:1	Used
**Sugars (1/1)**			
186	H1	Hexose	Used
