# Supplementary material for: Association of Metabolites with Obesity and Type 2 Diabetes Based on FTO Genotype
Source: PLoS One. 2016 Jun 1;11(6):e0156612. doi: 10.1371/journal.pone.0156612 (PMC4889059; doi:10.1371/journal.pone.0156612)
Supplement: S2 Table — Significant association defined by Benjamini-Hochberge adjusted p < 0.05.a (PDF) [file pone.0156612.s003.pdf]

S2 Table. Identified metabolites association with carriers of *FTO* rs9939609 (TA/AA genotype) by using dominant model. Significant association defined by Benjamini-Hochberge adjusted $p < 0.05$.[a]

	Metabolite	β	95 % CI	adjusted *p*-value
1	H1	0.126	0.04 - 0.22	4.6.E-02
2	Val	0.127	0.04 - 0.22	3.9.E-02
3	PC aa C36:5	0.175	0.08 - 0.27	1.2.E-02
4	PC aa C36:6	0.145	0.06 - 0.23	1.7.E-02
5	PC aa C38:0	0.128	0.04 - 0.22	3.9.E-02
6	PC aa C38:1	0.157	0.07 - 0.25	1.5.E-02
7	PC aa C38:5	0.140	0.05 - 0.23	2.8.E-02
8	PC aa C38:6	0.154	0.07 - 0.24	1.5.E-02
9	PC aa C40:1	0.123	0.03 - 0.21	4.8.E-02
10	PC aa C40:6	0.173	0.08 - 0.26	1.2.E-02
11	PC aa C42:2	0.145	0.06 - 0.23	1.5.E-02
12	PC aa C42:6	0.137	0.05 - 0.23	3.1.E-02
13	PC ae C34:0	0.165	0.07 - 0.25	1.5.E-02
14	PC ae C36:0	0.146	0.06 - 0.23	1.5.E-02
15	PC ae C38:0	0.134	0.04 - 0.22	3.1.E-02
16	PC ae C38:6	0.157	0.07 - 0.25	1.5.E-02
17	PC ae C40:2	0.129	0.04 - 0.22	3.9.E-02
18	PC ae C40:5	0.147	0.06 - 0.23	1.5.E-02
19	PC ae C40:6	0.147	0.06 - 0.24	1.5.E-02

aa, diacyl; ae, acyl-alkyl; PC, phosphatidlycholine.
