# Supplementary material for: Association of Metabolites with Obesity and Type 2 Diabetes Based on FTO Genotype
Source: PLoS One. 2016 Jun 1;11(6):e0156612. doi: 10.1371/journal.pone.0156612 (PMC4889059; doi:10.1371/journal.pone.0156612)
Supplement: S3 Table — (PDF) [file pone.0156612.s004.pdf]

S3 Table. Identified metabolites association with *FTO* genotype, 40 homozygotes with two risk alleles (AA), 570 heterozygous carriers (AT), and 1,967 homozygotes carrying no risk allele (TT), by using additive model.

**#**	**Metabolite**	**β**	**95 % CI**	**adjusted *p*-value**
1	PC aa C36:5	0.156	0.07-0.24	1.1.E-02
2	PC aa C40:6	0.154	0.07-0.24	1.1.E-02
3	PC ae C34:0	0.15	0.07-0.23	1.1.E-02
4	PC ae C38:6	0.148	0.07-0.23	1.1.E-02
5	PC aa C38:1	0.146	0.06-0.23	1.2.E-02
6	PC ae C36:0	0.137	0.06-0.22	1.5.E-02
7	PC aa C42:2	0.133	0.05-0.21	1.6.E-02
8	PC ae C40:5	0.133	0.05-0.21	1.6.E-02
9	PC ae C40:6	0.135	0.05-0.22	1.6.E-02
10	PC aa C38:6	0.132	0.05-0.21	1.8.E-02
11	PC aa C42:6	0.131	0.05-0.21	2.3.E-02
12	PC aa C36:6	0.126	0.05-0.21	2.5.E-02
13	PC aa C38:5	0.124	0.04-0.21	3.2.E-02
14	PC aa C38:0	0.117	0.04-0.2	4.0.E-02
15	H1	0.113	0.03-0.2	5.2.E-02
16	Val	0.112	0.03-0.19	5.2.E-02
17	PC aa C40:1	0.11	0.03-0.19	5.2.E-02
18	PC ae C38:0	0.11	0.03-0.19	5.2.E-02
19	PC ae C40:2	0.113	0.03-0.19	5.2.E-02
20	PC ae C44:4	-0.112	-0.19--0.03	5.2.E-02
