# Supplementary material for: Association of Metabolites with Obesity and Type 2 Diabetes Based on FTO Genotype
Source: PLoS One. 2016 Jun 1;11(6):e0156612. doi: 10.1371/journal.pone.0156612 (PMC4889059; doi:10.1371/journal.pone.0156612)
Supplement: S4 Table — (PDF) [file pone.0156612.s005.pdf]

S4 Table. Identified metabolites association with risk of obesity (BMI) in KARE S2 (Significant association defined by Benjamini-Hochberge adjusted $p < 0.05$).[a]

	**Metabolite**	**β**	**95 % CI**	**adjusted *p*-value**
1	Ac-Orn	-11.956	-14.51 - -9.4	8.9.E-19
2	ADMA	2.873	0.31 - 5.44	3.7.E-02
3	Ala	14.893	12.37 - 17.42	8.9.E-29
4	Asn	-7.957	-10.51 - -5.4	3.6.E-09
5	C14:1	10.787	8.17 - 13.4	4.8.E-15
6	C14:2	8.170	5.56 - 10.78	3.1.E-09
7	C16	15.232	12.65 - 17.81	8.9.E-29
8	C18	9.869	7.13 - 12.61	8.6.E-12
9	C18:1	8.838	6.15 - 11.52	4.2.E-10
10	C2	7.851	5.28 - 10.43	7.7.E-09
11	C3	4.106	1.5 - 6.71	3.4.E-03
12	C5	11.673	8.99 - 14.36	2.1.E-16
13	C7-DC	3.958	1.31 - 6.61	5.5.E-03
14	C8	10.278	7.71 - 12.85	3.5.E-14
15	Cit	-8.805	-11.51 - -6.1	6.4.E-10
16	Creatinine	-7.294	-10.37 - -4.22	7.6.E-06
17	Gln	-7.320	-9.85 - -4.79	4.5.E-08
18	Glu	10.877	8.32 - 13.44	8.4.E-16
19	Gly	-16.070	-18.59 - -13.55	3.9.E-33
20	H1	42.077	39.76 - 44.39	1.2.E-222
21	His	-3.382	-5.96 - -0.8	1.5.E-02
22	Ile	9.573	6.84 - 12.31	3.1.E-11
23	Leu	8.720	6.02 - 11.42	8.7.E-10
24	Lys	2.821	0.24 - 5.4	4.2.E-02
25	Phe	5.827	3.24 - 8.41	2.1.E-05
26	Pro	6.105	3.44 - 8.77	1.6.E-05
27	Sarcosine	-4.591	-7.35 - -1.83	1.9.E-03
28	Ser	-3.297	-5.85 - -0.75	1.7.E-02
29	Serotonin	-3.716	-6.3 - -1.13	7.5.E-03
30	Spermine	-4.932	-7.53 - -2.34	3.7.E-04
31	Thr	-4.130	-6.76 - -1.5	3.5.E-03
32	Trp	-3.483	-6.14 - -0.82	1.5.E-02
33	Tyr	3.714	1.11 - 6.32	8.0.E-03
34	Val	14.714	12.13 - 17.3	4.7.E-27
35	lysoPC a C16:0	5.725	3.19 - 8.26	2.0.E-05
36	lysoPC a C17:0	-10.489	-13.01 - -7.97	3.0.E-15
37	lysoPC a C18:0	-2.931	-5.47 - -0.39	3.3.E-02
38	lysoPC a C18:1	-7.175	-9.76 - -4.59	1.5.E-07
39	lysoPC a C18:2	-17.545	-20.11 - -14.98	8.0.E-38

40	lysoPC a C20:4	-2.997	-5.6 - -0.39	3.3.E-02
41	PC aa C28:1	6.931	4.36 - 9.5	3.4.E-07
42	PC aa C32:0	6.174	3.6 - 8.75	6.4.E-06
43	PC aa C32:1	8.655	6.05 - 11.26	3.3.E-10
44	PC aa C34:1	11.270	8.7 - 13.84	1.1.E-16
45	PC aa C34:2	9.642	7.09 - 12.19	7.8.E-13
46	PC aa C34:4	5.432	2.89 - 7.97	5.7.E-05
47	PC aa C36:0	-2.837	-5.45 - -0.22	4.3.E-02
48	PC aa C36:1	7.217	4.65 - 9.78	9.9.E-08
49	PC aa C36:2	4.781	2.22 - 7.34	4.6.E-04
50	PC aa C36:3	4.652	2.09 - 7.21	6.6.E-04
51	PC aa C36:4	7.510	4.98 - 10.04	2.0.E-08
52	PC aa C36:5	10.261	7.75 - 12.77	8.3.E-15
53	PC aa C36:6	5.157	2.57 - 7.75	1.8.E-04
54	PC aa C38:0	-3.007	-5.64 - -0.37	3.4.E-02
55	PC aa C38:1	-3.807	-6.39 - -1.22	6.1.E-03
56	PC aa C38:3	4.933	2.29 - 7.57	4.6.E-04
57	PC aa C38:5	8.280	5.77 - 10.79	4.2.E-10
58	PC aa C38:6	12.642	10.1 - 15.19	5.6.E-21
59	PC aa C40:1	-5.447	-8.02 - -2.87	6.6.E-05
60	PC aa C40:2	-3.177	-5.72 - -0.63	2.1.E-02
61	PC aa C40:3	-3.290	-5.84 - -0.74	1.7.E-02
62	PC aa C40:5	7.320	4.75 - 9.9	7.6.E-08
63	PC aa C40:6	9.786	7.26 - 12.32	2.1.E-13
64	PC aa C42:0	-10.894	-13.46 - -8.33	8.4.E-16
65	PC aa C42:1	-9.766	-12.36 - -7.17	9.2.E-13
66	PC aa C42:5	6.706	4.18 - 9.24	5.4.E-07
67	PC ae C32:1	-4.001	-6.56 - -1.44	3.6.E-03
68	PC ae C32:2	-3.896	-6.48 - -1.32	5.0.E-03
69	PC ae C34:1	-6.021	-8.59 - -3.45	1.0.E-05
70	PC ae C34:2	-10.998	-13.55 - -8.45	3.1.E-16
71	PC ae C34:3	-14.472	-16.98 - -11.97	1.1.E-27
72	PC ae C36:0	6.480	3.85 - 9.11	3.4.E-06
73	PC ae C36:2	-8.883	-11.43 - -6.33	3.9.E-11
74	PC ae C36:3	-12.880	-15.4 - -10.36	3.9.E-22
75	PC ae C36:4	-3.786	-6.37 - -1.2	6.5.E-03
76	PC ae C38:0	3.134	0.54 - 5.73	2.5.E-02
77	PC ae C38:1	3.093	0.5 - 5.68	2.7.E-02
78	PC ae C38:3	-3.139	-5.72 - -0.56	2.4.E-02
79	PC ae C38:4	-6.654	-9.21 - -4.1	8.4.E-07
80	PC ae C38:5	-4.219	-6.8 - -1.63	2.4.E-03
81	PC ae C40:1	-5.938	-8.54 - -3.34	1.7.E-05
82	PC ae C40:2	-2.879	-5.44 - -0.32	3.7.E-02
83	PC ae C40:3	-5.525	-8.1 - -2.95	5.4.E-05
84	PC ae C40:4	-5.521	-8.12 - -2.92	6.1.E-05

85	PC ae C40:5	4.935	2.3 - 7.57	4.4.E-04
86	PC ae C42:0	-9.124	-11.65 - -6.6	6.7.E-12
87	PC ae C42:1	-12.406	-14.92 - -9.89	8.6.E-21
88	PC ae C42:2	-3.856	-6.42 - -1.29	5.2.E-03
89	PC ae C42:3	-6.094	-8.68 - -3.5	9.1.E-06
90	PC ae C42:4	-11.385	-13.92 - -8.85	1.9.E-17
91	PC ae C42:5	-6.689	-9.25 - -4.13	7.9.E-07
92	PC ae C44:3	-6.825	-9.35 - -4.3	3.3.E-07
93	PC ae C44:4	-12.049	-14.57 - -9.53	1.5.E-19
94	PC ae C44:5	-9.905	-12.42 - -7.39	7.9.E-14
95	PC ae C44:6	-12.545	-15.07 - -10.02	5.6.E-21
96	SM (OH) C14:1	-9.443	-12.12 - -6.77	2.1.E-11
97	SM (OH) C16:1	-10.313	-12.95 - -7.68	1.1.E-13
98	SM (OH) C22:2	-15.683	-18.48 - -12.89	2.4.E-26
99	SM (OH) C24:1	-6.858	-9.44 - -4.27	5.3.E-07
100	SM C16:0	-13.621	-16.21 - -11.04	2.3.E-23
101	SM C16:1	-10.596	-13.43 - -7.76	1.2.E-12
102	SM C18:1	-6.007	-8.83 - -3.19	6.0.E-05
103	SM C24:1	-10.660	-13.19 - -8.13	1.3.E-15
104	SM C26:1	-10.742	-13.24 - -8.24	4.2.E-16

[a], acyl; aa, diacyl; ae, acyl-alkyl; PC, phosphatidlycholine; SM, sphingomyelin.
