# Supplementary material for: Association of Metabolites with Obesity and Type 2 Diabetes Based on FTO Genotype
Source: PLoS One. 2016 Jun 1;11(6):e0156612. doi: 10.1371/journal.pone.0156612 (PMC4889059; doi:10.1371/journal.pone.0156612)
Supplement: S5 Table — (PDF) [file pone.0156612.s006.pdf]

S5 Table. Identified metabolites association with risk of **T2D (Glu0)** in KARE S2 (Significant association defined by Benjamini-Hochberge adjusted $p < 0.05$).[a]

	**Metabolite**	**β-coefficient**	**95% CI**	***p* value**
1	C14:1	1.81	1.01 - 2.62	2.3E-05
2	C16	3.09	2.29 - 3.89	2.2E-13
3	C18	2.16	1.32 - 3.00	1.3E-06
4	C18:1	2.21	1.39 - 3.03	4.2E-07
5	C3	1.95	1.16 - 2.74	4.0E-06
6	C5	3.19	2.37 - 4.01	2.1E-13
7	Ala	4.44	3.67 - 5.2	2.6E-27
8	Asn	-2.15	-2.93 - -1.37	2.2E-07
9	Cit	-2.48	-3.31 - -1.66	1.2E-08
10	Gln	-3.85	-4.61 - -3.09	1.5E-21
11	Glu	3.17	2.39 - 3.95	1.5E-14
12	Gly	-4.07	-4.84 - -3.3	4.1E-23
13	H1	16.68	16.12 - 17.24	0.0E+00
14	His	-1.72	-2.51 - -0.94	3.9E-05
15	Ile	2.41	1.58 - 3.25	5.3E-08
16	Leu	2.14	1.32 - 2.96	1.1E-06
17	Phe	1.45	0.67 - 2.24	5.7E-04
18	Pro	1.81	0.99 - 2.62	3.1E-05
19	Trp	-1.74	-2.55 - -0.93	5.3E-05
20	Tyr	1.48	0.69 - 2.27	4.9E-04
21	Val	3.74	2.95 - 4.53	5.7E-19
22	Ac-Orn	-3.51	-4.29 - -2.73	1.8E-17
23	Creatinine	-3.44	-4.36 - -2.51	2.5E-12
24	Kynurenine	-1.02	-1.82 - -0.22	2.0E-02
25	Serotonin	-1.32	-2.11 - -0.54	1.8E-03
26	Spermine	-0.87	-1.66 - -0.08	4.5E-02
27	lysoPC a C16:0	2.75	1.99 - 3.52	9.5E-12
28	lysoPC a C16:1	1.07	0.29 - 1.86	1.3E-02
29	lysoPC a C17:0	-1.62	-2.39 - -0.84	9.2E-05
30	lysoPC a C18:2	-3.67	-4.47 - -2.87	3.6E-18
31	lysoPC a C20:4	1.07	0.28 - 1.86	1.3E-02
32	PC aa C28:1	1.39	0.6 - 2.17	1.0E-03
33	PC aa C32:0	1.91	1.12 - 2.69	5.1E-06
34	PC aa C32:1	2.05	1.25 - 2.85	1.4E-06
35	PC aa C34:1	3.02	2.24 - 3.81	2.9E-13
36	PC aa C34:2	2.84	2.06 - 3.62	4.3E-12
37	PC aa C34:4	2.62	1.85 - 3.38	1.2E-10
38	PC aa C36:0	-0.96	-1.75 - -0.16	2.9E-02
39	PC aa C36:1	2.04	1.26 - 2.82	9.5E-07
40	PC aa C36:2	1.84	1.06 - 2.62	9.4E-06

41	PC aa C36:3	1.22	0.44 - 2	3.8E-03
42	PC aa C36:4	2.85	2.09 - 3.62	1.9E-12
43	PC aa C36:5	3.46	2.70 - 4.22	1.1E-17
44	PC aa C36:6	1.72	0.93 - 2.50	4.4E-05
45	PC aa C38:0	-0.94	-1.74 - -0.14	3.3E-02
46	PC aa C38:4	1.02	0.25 - 1.79	1.5E-02
47	PC aa C38:5	3.00	2.23 - 3.76	1.1E-13
48	PC aa C38:6	2.81	2.03 - 3.59	9.5E-12
49	PC aa C40:1	-1.69	-2.48 - -0.91	5.1E-05
50	PC aa C40:2	-0.87	-1.64 - -0.09	4.3E-02
51	PC aa C40:4	-0.86	-1.64 - -0.07	4.6E-02
52	PC aa C40:5	2.07	1.28 - 2.85	7.7E-07
53	PC aa C40:6	1.77	0.99 - 2.54	2.0E-05
54	PC aa C42:0	-3.31	-4.09 - -2.52	1.3E-15
55	PC aa C42:1	-3.13	-3.92 - -2.34	6.1E-14
56	PC aa C42:4	-1.30	-2.07 - -0.53	1.8E-03
57	PC aa C42:5	2.25	1.48 - 3.02	3.8E-08
58	PC ae C30:0	-1.23	-2.02 - -0.44	4.2E-03
59	PC ae C32:1	-0.96	-1.74 - -0.18	2.5E-02
60	PC ae C32:2	-1.07	-1.86 - -0.29	1.3E-02
61	PC ae C34:1	-1.54	-2.32 - -0.76	2.4E-04
62	PC ae C34:2	-2.57	-3.35 - -1.79	4.8E-10
63	PC ae C34:3	-4.13	-4.89 - -3.36	3.9E-24
64	PC ae C36:2	-1.64	-2.42 - -0.86	8.1E-05
65	PC ae C36:3	-3.25	-4.02 - -2.48	1.6E-15
66	PC ae C36:5	0.88	0.09 - 1.67	4.3E-02
67	PC ae C38:0	0.96	0.17 - 1.75	2.7E-02
68	PC ae C38:3	-1.11	-1.9 - -0.33	9.7E-03
69	PC ae C38:4	-1.11	-1.89 - -0.33	9.7E-03
70	PC ae C40:1	-1.94	-2.73 - -1.15	4.3E-06
71	PC ae C40:3	-2.01	-2.79 - -1.23	1.4E-06
72	PC ae C40:4	-1.72	-2.51 - -0.93	4.6E-05
73	PC ae C40:5	1.88	1.08 - 2.68	9.8E-06
74	PC ae C40:6	-0.88	-1.67 - -0.08	4.5E-02
75	PC ae C42:0	-2.55	-3.32 - -1.78	3.7E-10
76	PC ae C42:1	-3.29	-4.06 - -2.52	6.2E-16
77	PC ae C42:2	-1.47	-2.25 - -0.69	4.6E-04
78	PC ae C42:3	-1.70	-2.48 - -0.91	5.5E-05
79	PC ae C42:4	-3.50	-4.27 - -2.73	1.1E-17
80	PC ae C42:5	-1.41	-2.19 - -0.63	8.0E-04
81	PC ae C44:3	-1.86	-2.63 - -1.09	5.5E-06
82	PC ae C44:4	-4.11	-4.88 - -3.35	3.9E-24
83	PC ae C44:5	-2.18	-2.95 - -1.41	1.0E-07
84	PC ae C44:6	-3.75	-4.52 - -2.99	3.7E-20
85	SM (OH) C14:1	-3.44	-4.25 - -2.63	1.1E-15

86	SM (OH) C16:1	-3.37	-4.17 - -2.57	1.6E-15
87	SM (OH) C22:2	-5.21	-6.05 - -4.36	8.8E-31
88	SM (OH) C24:1	-1.08	-1.87 - -0.29	1.3E-02
89	SM C16:0	-3.78	-4.57 - -2.99	2.1E-19
90	SM C16:1	-3.31	-4.17 - -2.45	3.2E-13
91	SM C18:1	-2.81	-3.67 - -1.96	4.8E-10
92	SM C24:1	-3.18	-3.94 - -2.4	6.5E-15
93	SM C26:1	-2.95	-3.71 - -2.19	2.6E-13

[a] a, acyl; aa, diacyl; ae, acyl-alkyl; PC, phosphatidlycholine; SM, sphingomyelin; CI, confidence interval.
