# Supplementary material for: Association of Metabolites with Obesity and Type 2 Diabetes Based on FTO Genotype
Source: PLoS One. 2016 Jun 1;11(6):e0156612. doi: 10.1371/journal.pone.0156612 (PMC4889059; doi:10.1371/journal.pone.0156612)
Supplement: S6 Table — (PDF) [file pone.0156612.s007.pdf]

**S6 Table**. Identified metabolites association with risk of **T2D (Glu120)** in KARE S2 (Significant association defined by Benjamini-Hochberge adjusted $p < 0.05$).[a]

| | **Metabolite** | **β-coefficient** | **95% CI** | ***p* value** |
|---|---|---|---|---|
| 1 | Ac-Orn | -11.96 | -14.51 - -9.40 | 8.9E-19 |
| 2 | ADMA | 2.87 | 0.31 - 5.44 | 3.7E-02 |
| 3 | Ala | 14.89 | 12.37 - 17.42 | 8.9E-29 |
| 4 | Asn | -7.96 | -10.51 - -5.40 | 3.6E-09 |
| 5 | C14:1 | 10.79 | 8.17 - 13.40 | 4.8E-15 |
| 6 | C14:2 | 8.17 | 5.56 - 10.78 | 3.1E-09 |
| 7 | C16 | 15.23 | 12.65 - 17.81 | 8.9E-29 |
| 8 | C18 | 9.87 | 7.13 - 12.61 | 8.6E-12 |
| 9 | C18:1 | 8.84 | 6.15 - 11.52 | 4.2E-10 |
| 10 | C2 | 7.85 | 5.28 - 10.43 | 7.7E-09 |
| 11 | C3 | 4.11 | 1.50 - 6.71 | 3.4E-03 |
| 12 | C5 | 11.67 | 8.99 - 14.36 | 2.1E-16 |
| 13 | C7-DC | 3.96 | 1.31 - 6.61 | 5.5E-03 |
| 14 | C8 | 10.28 | 7.71 - 12.85 | 3.5E-14 |
| 15 | Cit | -8.81 | -11.51 - -6.10 | 6.4E-10 |
| 16 | Creatinine | -7.29 | -10.37 - -4.22 | 7.6E-06 |
| 17 | Gln | -7.32 | -9.85 - -4.79 | 4.5E-08 |
| 18 | Glu | 10.88 | 8.32 - 13.44 | 8.4E-16 |
| 19 | Gly | -16.07 | -18.59 - -13.55 | 3.9E-33 |
| 20 | H1 | 42.08 | 39.76 - 44.39 | 1.2E-222 |
| 21 | His | -3.38 | -5.96 - -0.80 | 1.5E-02 |
| 22 | Ile | 9.57 | 6.84 - 12.31 | 3.1E-11 |
| 23 | Leu | 8.72 | 6.02 - 11.42 | 8.7E-10 |
| 24 | Lys | 2.82 | 0.24 - 5.40 | 4.2E-02 |
| 25 | Phe | 5.83 | 3.24 - 8.41 | 2.1E-05 |
| 26 | Pro | 6.11 | 3.44 - 8.77 | 1.6E-05 |
| 27 | Sarcosine | -4.59 | -7.35 - -1.83 | 1.9E-03 |
| 28 | Ser | -3.30 | -5.85 - -0.75 | 1.7E-02 |
| 29 | Serotonin | -3.72 | -6.30 - -1.13 | 7.5E-03 |
| 30 | Spermine | -4.93 | -7.53 - -2.34 | 3.7E-04 |
| 31 | Thr | -4.13 | -6.76 - -1.50 | 3.5E-03 |
| 32 | Trp | -3.48 | -6.14 - -0.82 | 1.5E-02 |
| 33 | Tyr | 3.71 | 1.11 - 6.32 | 8.0E-03 |
| 34 | Val | 14.71 | 12.13 - 17.30 | 4.7E-27 |
| 35 | lysoPC a C16:0 | 5.73 | 3.19 - 8.26 | 2.0E-05 |
| 36 | lysoPC a C17:0 | -10.49 | -13.01 - -7.97 | 3.0E-15 |
| 37 | lysoPC a C18:0 | -2.93 | -5.47 - -0.39 | 3.3E-02 |
| 38 | lysoPC a C18:1 | -7.18 | -9.76 - -4.59 | 1.5E-07 |
| 39 | lysoPC a C18:2 | -17.55 | -20.11 - -14.98 | 8.0E-38 |
| 40 | lysoPC a C20:4 | -3.00 | -5.60 - -0.39 | 3.3E-02 |

| | | | | |
|---|---|---|---|---|
| 41 | PC aa C28:1 | 6.93 | 4.36 - 9.50 | 3.4E-07 |
| 42 | PC aa C32:0 | 6.17 | 3.60 - 8.75 | 6.4E-06 |
| 43 | PC aa C32:1 | 8.66 | 6.05 - 11.26 | 3.3E-10 |
| 44 | PC aa C34:1 | 11.27 | 8.70 - 13.84 | 1.1E-16 |
| 45 | PC aa C34:2 | 9.64 | 7.09 - 12.19 | 7.8E-13 |
| 46 | PC aa C34:4 | 5.43 | 2.89 - 7.97 | 5.7E-05 |
| 47 | PC aa C36:0 | -2.84 | -5.45 - -0.22 | 4.3E-02 |
| 48 | PC aa C36:1 | 7.22 | 4.65 - 9.78 | 9.9E-08 |
| 49 | PC aa C36:2 | 4.78 | 2.22 - 7.34 | 4.6E-04 |
| 50 | PC aa C36:3 | 4.65 | 2.09 - 7.21 | 6.6E-04 |
| 51 | PC aa C36:4 | 7.51 | 4.98 - 10.04 | 2.0E-08 |
| 52 | PC aa C36:5 | 10.26 | 7.75 - 12.77 | 8.3E-15 |
| 53 | PC aa C36:6 | 5.16 | 2.57 - 7.75 | 1.8E-04 |
| 54 | PC aa C38:0 | -3.01 | -5.64 - -0.37 | 3.4E-02 |
| 55 | PC aa C38:1 | -3.81 | -6.39 - -1.22 | 6.1E-03 |
| 56 | PC aa C38:3 | 4.93 | 2.29 - 7.57 | 4.6E-04 |
| 57 | PC aa C38:5 | 8.28 | 5.77 - 10.79 | 4.2E-10 |
| 58 | PC aa C38:6 | 12.64 | 10.10 - 15.19 | 5.6E-21 |
| 59 | PC aa C40:1 | -5.45 | -8.02 - -2.87 | 6.6E-05 |
| 60 | PC aa C40:2 | -3.18 | -5.72 - -0.63 | 2.1E-02 |
| 61 | PC aa C40:3 | -3.29 | -5.84 - -0.74 | 1.7E-02 |
| 62 | PC aa C40:5 | 7.32 | 4.75 - 9.90 | 7.6E-08 |
| 63 | PC aa C40:6 | 9.79 | 7.26 - 12.32 | 2.1E-13 |
| 64 | PC aa C42:0 | -10.89 | -13.46 - -8.33 | 8.4E-16 |
| 65 | PC aa C42:1 | -9.77 | -12.36 - -7.17 | 9.2E-13 |
| 66 | PC aa C42:5 | 6.71 | 4.18 - 9.24 | 5.4E-07 |
| 67 | PC ae C32:1 | -4.00 | -6.56 - -1.44 | 3.6E-03 |
| 68 | PC ae C32:2 | -3.90 | -6.48 - -1.32 | 5.0E-03 |
| 69 | PC ae C34:1 | -6.02 | -8.59 - -3.45 | 1.0E-05 |
| 70 | PC ae C34:2 | -11.00 | -13.55 - -8.45 | 3.1E-16 |
| 71 | PC ae C34:3 | -14.47 | -16.98 - -11.97 | 1.1E-27 |
| 72 | PC ae C36:0 | 6.48 | 3.85 - 9.11 | 3.4E-06 |
| 73 | PC ae C36:2 | -8.88 | -11.43 - -6.33 | 3.9E-11 |
| 74 | PC ae C36:3 | -12.88 | -15.40 - -10.36 | 3.9E-22 |
| 75 | PC ae C36:4 | -3.79 | -6.37 - -1.20 | 6.5E-03 |
| 76 | PC ae C38:0 | 3.13 | 0.54 - 5.73 | 2.5E-02 |
| 77 | PC ae C38:1 | 3.09 | 0.50 - 5.68 | 2.7E-02 |
| 78 | PC ae C38:3 | -3.14 | -5.72 - -0.56 | 2.4E-02 |
| 79 | PC ae C38:4 | -6.65 | -9.21 - -4.10 | 8.4E-07 |
| 80 | PC ae C38:5 | -4.22 | -6.80 - -1.63 | 2.4E-03 |
| 81 | PC ae C40:1 | -5.94 | -8.54 - -3.34 | 1.7E-05 |
| 82 | PC ae C40:2 | -2.88 | -5.44 - -0.32 | 3.7E-02 |
| 83 | PC ae C40:3 | -5.53 | -8.10 - -2.95 | 5.4E-05 |
| 84 | PC ae C40:4 | -5.52 | -8.12 - -2.92 | 6.1E-05 |
| 85 | PC ae C40:5 | 4.94 | 2.30 - 7.57 | 4.4E-04 |

| | | | | |
|---|---|---|---|---|
| 86 | PC ae C42:0 | -9.12 | -11.65 - -6.60 | 6.7E-12 |
| 87 | PC ae C42:1 | -12.41 | -14.92 - -9.89 | 8.6E-21 |
| 88 | PC ae C42:2 | -3.86 | -6.42 - -1.29 | 5.2E-03 |
| 89 | PC ae C42:3 | -6.09 | -8.68 - -3.50 | 9.1E-06 |
| 90 | PC ae C42:4 | -11.39 | -13.92 - -8.85 | 1.9E-17 |
| 91 | PC ae C42:5 | -6.69 | -9.25 - -4.13 | 7.9E-07 |
| 92 | PC ae C44:3 | -6.83 | -9.35 - -4.30 | 3.3E-07 |
| 93 | PC ae C44:4 | -12.05 | -14.57 - -9.53 | 1.5E-19 |
| 94 | PC ae C44:5 | -9.91 | -12.42 - -7.39 | 7.9E-14 |
| 95 | PC ae C44:6 | -12.55 | -15.07 - -10.02 | 5.6E-21 |
| 96 | SM (OH) C14:1 | -9.44 | -12.12 - -6.77 | 2.1E-11 |
| 97 | SM (OH) C16:1 | -10.31 | -12.95 - -7.68 | 1.1E-13 |
| 98 | SM (OH) C22:2 | -15.68 | -18.48 - -12.89 | 2.4E-26 |
| 99 | SM (OH) C24:1 | -6.86 | -9.44 - -4.27 | 5.3E-07 |
| 100 | SM C16:0 | -13.62 | -16.21 - -11.04 | 2.3E-23 |
| 101 | SM C16:1 | -10.60 | -13.43 - -7.76 | 1.2E-12 |
| 102 | SM C18:1 | -6.01 | -8.83 - -3.19 | 6.0E-05 |
| 103 | SM C24:1 | -10.66 | -13.19 - -8.13 | 1.3E-15 |
| 104 | SM C26:1 | -10.74 | -13.24 - -8.24 | 4.2E-16 |

[a] a, acyl; aa, diacyl; ae, acyl-alkyl; PC, phosphatidlycholine; SM, sphingomyelin; CI, confidence interval.
